# Working hours and health in nurses of public hospitals according to gender

**DOI:** 10.1590/S1518-8787.2017051006808

**Published:** 2017-06-20

**Authors:** Juliana da Costa Fernandes, Luciana Fernandes Portela, Rosane Härter Griep, Lúcia Rotenberg

**Affiliations:** I Programa de Pós-Graduação em Saúde Pública. Escola Nacional de Saúde Pública. Fundação Oswaldo Cruz. Rio de Janeiro, RJ, Brasil; II Laboratório de Educação em Ambiente e Saúde. Instituto Oswaldo Cruz. Fundação Oswaldo Cruz. Rio de Janeiro, RJ, Brasil

**Keywords:** Nurses, Self-Assessment, Health Status, Work Hours, Working Conditions, Hospitals, Public

## Abstract

**OBJECTIVE:**

To assess the association between weekly working hours and self-rated health of nurses in public hospitals in Rio de Janeiro, State of Rio de Janeiro, Brazil.

**METHODS:**

A total of 3,229 nurses (82.7% of the eligible group) participated in this cross-sectional study, carried out between April 2010 and December 2011. The collection instrument consisted of a self-administered multidimensional questionnaire. The weekly working hours were calculated from a recall of the daily hours worked over seven consecutive days; this variable was categorized according to tertiles of distribution for men and women. The outcome of interest, self-rated health, was categorized into three levels: good (very good and good), regular, and poor (poor and very poor). The statistical analysis of the data included bivariate and multivariate analyses, having as reference group those with short working hours (first tertile). All the analyses were stratified by gender and elaborated using the program SPSS.

**RESULTS:**

Among women, the group corresponding to the longest working week (more than 60.5 hours per week) were more likely to report regular self-rated health, compared with those with shorter working hours, after adjusting for confounding factors (OR = 1.30; 95%CI 1.02–1.67). Among men, those with average working hours (49.5–70.5 hours per week) were more than twice as likely to rate their health as regular (OR = 2.17; 95%CI 1.08–4.35) compared to those with shorter working hours (up to 49.5 hours). There was no significant association between long working hours and poor self-rated health.

**CONCLUSIONS:**

The results point to the urgent need to promote interventions in the organization of work and appreciation of the nursing profession, in order to reduce the number of multiple jobs and thus contribute to mitigate potential effects on the health of workers and the quality of care in hospitals.

## RESUMO

**OBJETIVO:**

Avaliar a associação entre horas de trabalho semanais e autoavaliação de saúde de enfermeiros em hospitais públicos do Rio de Janeiro, RJ, Brasil.

**MÉTODOS:**

Um total de 3.229 enfermeiros (82,7% do grupo de elegíveis) participou deste estudo transversal, realizado entre abril de 2010 e dezembro de 2011. O instrumento de coleta consistiu em um questionário multidimensional autopreenchido. As horas de trabalho semanais foram calculadas a partir de um recordatório das horas diárias de trabalho ao longo de sete dias consecutivos; esta variável foi categorizada de acordo com tercis da distribuição para homens e mulheres. O desfecho de interesse, auto-avaliação de saúde, foi categorizado em três níveis: bom (muito bom e bom), regular e ruim (ruim e muito ruim). A análise estatística dos dados incluiu análises bivaridas e multivariadas, tendo como grupo de referência aqueles com jornadas curtas de trabalho (primeiro tercil). Todas as análises foram estratificadas por sexo e elaboradas no programa SPSS.

**RESULTADOS:**

Entre as mulheres, o grupo correspondente à semana de trabalho mais longa (mais de 60,5 horas por semana) tinha maior probabilidade de relatar autoavaliação de saúde como regular, em comparação com aqueles com jornada curta, após o ajuste para fatores de confusão (OR = 1,30; IC95% 1,02–1,67). Entre os homens, aqueles com jornada média (49,5-70,5 horas por semana) tiveram mais que o dobro da probabilidade de avaliar sua saúde como regular (OR = 2,17; IC95% 1,08–4,35) em comparação com aqueles com a semana de trabalho mais curta (até 49,5 horas). Não houve associação significativa entre longas horas de trabalho e autoavaliação de saúde ruim.

**CONCLUSÕES:**

Os resultados apresentados apontam para a urgência em promover intervenções na organização do trabalho e valorização da profissão de enfermagem, de modo a reduzir o múltiplo vínculo e assim contribuir para mitigar possíveis efeitos sobre a saúde dos trabalhadores e a qualidade do atendimento nos hospitais.

## INTRODUCTION

Time spent at work is an essential component of occupational exposure^14^. Dal Rosso^6^ emphasizes the role of working hours in the contemporary debate on their implications to the health and quality of life of workers. We have to consider the increasing demands arising from the process of globalization that defy human limits, because “thanks to a life that is practically focused on work, which is generally intense and accelerated, we have also often noticed the appearance and establishment of psychophysiological and social impacts on a large scale on workers” (p. 284)^8^.

The contribution of the scientific community to this debate has intensified in recent years, most notably the conceptual framework^4^, which addresses the complexity of the relationships between the time dedicated to work and the health of workers. Epidemiological investigations in various occupational groups show the influence of long working hours on hypertension and metabolic syndrome, in addition to associations with coronary heart disease, sleep disorders, and states of depression and anxiety, as indicated in a recent systematic review^2^.

In Brazil, the nursing staff is an important group in the context of those discussions, given the long shifts (usually 12 hours) adopted in hospitals and the practice of multiple jobs, leading to the exacerbation of working hours^7,9^. Brazilian studies on working hours in this group have approached habits and behaviors, such as excessive consumption of fried foods and coffee, lack of physical activity, and increased prevalence of obesity^9^, and aspects of well-being, such as the non-availability of time for rest and leisure^25^ and recovery after work^23^. However, few investigations have assessed outcomes more directly linked to health. In this study, we used the self-rated health as an indicator of health status, which is considered as a consistent predictor of morbidity and mortality in epidemiological studies^28^.

The study seeks to test the hypothesis that professionals who dedicate more time to work have a higher chance of assessing their health as poor or regular, compared to those with shorter working hours. The aim of this study was to analyze the association between working hours and the self-rated health in nurses of public hospitals, considering possible gender differences.

## METHODS

### Participants and Data Collection

This is a census of the eighteen major public hospitals of the city of Rio de Janeiro, State of Rio de Janeiro, Brazil, carried out between March 2010 and December 2011^10^. From the lists of names and sectors of nurses working in all hospitals, we obtained a general list of the set of workers. We considered as not eligible the nurses who were on leave, those who were replaced by other professionals by personal agreement, and those transferred or dismissed^10^.

Data collection was based on a multidimensional and structured self-administered questionnaire^10^. A team of trained professionals was responsible for delivering the questionnaires to the nurses accompanied by an informed consent. After signing it, the participants were instructed to fill out the questionnaire, seal it, and return it to the research team at the scheduled time.

### Definition of Variables

#### Variable of exposure 

The definition of “working hours” refers to the total time spent to perform the activities of work during the seven days preceding the interview, encompassing all jobs, following the concept proposed by Dal Rosso^6^. The calculation of the weekly working hours was based on the question: “How many hours did you dedicate to the professional nursing work each day of the last week? Use the table below for a recall. In addition, consider overtime and work taken home”. The sum of the professional working hours reported by the interviewee generated a continuous variable named “working hours”, categorized according to the tertile of the distribution according to gender^5^. For the male group, we adopted the values “< 49.5 h/week”, “from 49.5h to 70.5h”, and “> 70.5 h/week” for short, average, and long working hours, respectively. For the women, the values adopted were “< 46.5 h/week”, “46.5h to 60.5h”, and “> 60.5 h/week”.

#### Outcome variable: self-rated health

The outcome of interest was assessed from the following question: “In general, compared to persons in your age, what is your health status?”. This question presented five response options: (1) Very good, (2) Good, (3) Regular, (4) Poor, and (5) Very poor. This variable was categorized into three levels: good (very good or good), regular (regular), and poor (poor or very poor).

## Covariates

We considered in this study the relevant covariates for both the exposure investigated and the outcome of interest, namely: (i) sociodemographic data: age (continuous), marital status (married/common-law marriage; single/no partner), self-reported race (white; non-white), education level (post-graduate; university degree), *per capita* income calculated based on the mid-point of income (up to R$1,394.83; R$1,394.90 to R$2,324.50; R$2,324.83 to R$7,440.00), and hours on household chores (continuous); (ii) data related to work: work shift (day and night), number of jobs (one; two or more), type of employment (public servant; outsourced), time working in nursing (continuous), thinking frequently about leaving the profession (no; yes)^13^, psychosocial stress based on the demand-control model^16^ (low strain; passive work; active work; high strain [based on quadrants]), social support at the workplace (high; low [based on the median]), and effort-reward imbalance (low; average; high [based on tertiles])^24^; (iii) variables related to health: practice of physical activity (yes; no), duration of sleep per night (up to 6.5h; from 7h to 8h; from 8.5 to 12h), smoking (non-smoker; former smoker; smoker), consumption of alcoholic beverages (never; up to four times a month; more than four times a month); and body mass index (BMI), defined by self-reported weight (kg)/height (m^2^). From the BMI, individuals were classified as eutrophic (< 24.99), overweight (25.00–29.99), or obese (≥ 30.00).

## Statistical Treatment of the Data

All the analyses were prepared separately for the male and female groups, taking into account previous studies that show important differences both in the variable of exposure^9^ and outcome^15^. The characterization of the sample as to the variables of exposure and outcome was based on bivariate statistical analyses using the Chi-square and ANOVA tests (p < 0.05).

The association between the long working hours and the self-rated health was analyzed in two steps. The first concerns the definition of confounding variables, based on bivariate analyses using the Chi-square and ANOVA tests. We tested as potential confounding factors all variables described earlier with the exception of the following variables: number of jobs; hours on household chores; time working in nursing; psychosocial stress, assessed according to the demand-control model; and thinking about leaving the profession. We considered as variables of adjustment all those that were associated both to the outcome and to exposure with a 20% significance level.

The second step refers to the multinomial logistic regression model, considering two outcomes: regular self-reported health and poor self-reported health. We adopted the following sequence of adjustments for both groups: model 1: adjusted for socio-demographic variables; model 2: model 1 + occupational variables; and model 3: model 2 + variables related to health.

## Ethical Procedures

The study was approved by the Research Ethics Committee of the Fundação Oswaldo Cruz (FIOCRUZ – Process 472/08), and subsequently approved by ethics committees of some of the hospitals studied. Some hospitals that had no committees accepted the approval by the Research Ethics Committee of FIOCRUZ.

## RESULTS

The sample studied had 3,229 nurses, which corresponds to 82.7% of the total eligible (n = 3,904). The losses are due to refusals (n = 478) and nurses that were not located in the hospitals over two months (n = 128). Of the sample studied, 2,818 (87.3%) were female.

The female group had an average age of 39.7 years (SD = 9.9 years). Approximately 55.7% self-reported as white; 55.9% were married or had a common-law marriage; and, 75.9% had a graduate degree. As for income, 40% of the female nurses were classified in the group corresponding to the per capita income of more than R$2,300.00. The average time spent with household chores was 21 hours per week, while average working hours was 55.1 hours per week (SD = 20.9 hours). Almost 50% of the nurses worked in night shifts and approximately 2/3 worked in two or more locations. As for the aspects related to health, 7.1% reported having poor or very poor health. Approximately half of the participants were overweight and 23.7% were smokers or former smokers.

The male group had an average age of 41.4 years (SD = 10.7 years). Approximately 58.7% self-reported as white; 68.3% were married or had a common-law marriage; and, 69.8% had a graduate degree. As for income, 40% of the male nurses were classified in the group corresponding to the per capita income of more than R$2,300.00. The average time spent with household chores was 12.7 hours per week (SD ± 12.0 hours), while average working hours was 61.3 hours per week (SD = 21.5 hours). Most male participants (63.1%) worked in night shifts and 79.1% reported having two or more jobs in the area of nursing. The poor or very poor assessment of health was reported by 6.8% of the male nurses. Almost 25% were smokers or former smokers and 69.7% did not practice physical activity.

The self-reported health data were similar between men and women, with percentages of good, regular, and poor self-rated health of 65.8%, 27.1%, and 7.1% among women and 65.5%, 27.6%, and 6.8% among men, respectively.

The bivariate analyses showed that women exposed to long working hours were younger (average of 38.3 years), worked for less time in nursing (14.0 years) showed reduced hours on household chores (average of 18.2 hours), had higher education, and more frequently reported the thought of leaving the profession, compared to those who had short working hours. Long working hours were significantly associated with night work, more jobs, and being outsourced. Among the workers who reported long working hours, we observed a higher proportion of those who reported short sleep and no practice of physical activity, compared to those who had short working hours. In relation to psychosocial stress at work, we observed a higher frequency of the nurses in the group for high effort-reward imbalance, high strain, and low social support among those who have long working hours, compared to those who had short working hours (Table 1).

**Table 1:** Characterization of the weekly working hours of the female group according to socio-demographic, occupational, and health-related variables. Rio de Janeiro, State of Rio de Janeiro, Brazil, 2011.

Characteristics studied	Weekly working hours			p*

	Short (< 46,5h)	Average (46,5–60,5h)	Long (> 60,5h)	
Age (average, SD)	41.8 (10.8)	38.9 (9.4)	38.3 (9.0)	< 0.001
Marital status (n, %)				
Married/Common-law marriage	504 (33.3)	516 (31.1)	492 (32.5)	0.536
Single/Without partner	393 (33.2)	383 (32.4)	406 (34.4)	
Education level (n, %)				
Post-graduate (*lato sensu* and *stricto sensu*)	635 (31.1)	668 (32.7)	741 (36.3)	< 0.001
University degree	259 (39.9)	231 (35.6)	159 (24.5)	
Household income *per capita* (n, %)				
Up to R$1,394.83	310 (31.9)	331 (34.1)	330 (34.0)	
R$1,394.90 to R$2,324.50	390 (32.4)	396 (32.9)	416 (34.6)	0.375
R$2,324.83 to R$7,440.00	132 (37.4)	113 (32.0)	108 (30.6)	
Hours on household chores (average, SD)	25.5 (20.0)	20.3 (16.1)	18.2 (15.4)	< 0.001
Time working in nursing (average, SD)	16.7 (10.83)	14.64 (9.11)	14.0 (8.5)	< 0.001
Work shift (n, %)				
Day	561 (41.0)	468 (34.2)	340 (24.8)	< 0.001
Night	300 (23.7)	420 (33.1)	548 (43.2)	
Number of jobs (n, %)				
One job	634 (67.4)	224 (23.8)	82 (9.0)	< 0.001
Two or more jobs	269 (15.1)	684 (38.5)	825 (46.4)	
Type of employment (n, %)				
Public servant	616 (34.0)	631 (34.8)	564 (31.1)	0.002
Outsourced	262 (31.4)	256 (30.7)	317 (38.0)	
Effort-reward imbalance (n, %)				
Low	358 (41.6)	282 (32.4)	216 (28.2)	
Average	289 (33.6)	288 (33.1)	300 (39.2)	< 0.001
High	213 (24.8)	301 (34.5)	250 (32.6)	
Demand-control (n, %)				
Low strain	282 (33.2)	230 (27.2)	190 (22.3)	
Passive work	221 (26.0)	170 (20.0)	155 (18.2)	< 0.001
Active work	155 (18.3)	223 (26.4)	258 (30.3)	
High strain	191 (22.5)	223 (26.4)	249 (29.2)	
Social support in the workplace (n, %)				
High	504 (57.3)	455 (51.2)	414 (46.8)	< 0.001
Low	376 (42.7)	434 (48.8)	470 (53.2)	
Thinking about leaving the profession (n, %)				
Not frequently	710 (79.1)	737 (81.5)	686 (76.2)	0.022
Frequently	188 (20.9)	167 (18.5)	214 (23.8)	
Practice of physical activity (n, %)				
Yes	297 (36.2)	273 (33.3)	250 (30.5)	0.043
No	597 (31.9)	627 (33.5)	650 (34.7)	
Duration of sleep per night (n, %)				
Up to 6.5h	407 (32.2)	415 (32.9)	441 (34.9)	
From 7 to 8h	406 (35.9)	372 (32.9)	352 (31.2)	0.039
From 8.5 to 12h	79 (27.7)	109 (38.2)	97 (34.0)	
Use of tobacco (n, %)				
Smoker	85 (36.3)	72 (30.8)	77 (32.9)	0.858
Former smoker	134 (33.4)	132 (32.9)	135 (33.7)	
Non-smoker	682 (32.9)	698 (33.7)	690 (33.3)	
Use of alcoholic beverages (n, %)				
Never	355 (34.0)	350 (33.5)	340 (32.5)	
Up to 4 times a month	452 (32.1)	473 (33.5)	485 (34.4)	0.489
More than 4 times a month	85 (37.3)	75 (32.9)	68 (29.8)	
Body mass index (n, %)				
Eutrophic	420 (49.2)	458 (52.9)	401 (46.4)	
Overweight	263 (30.8)	249 (28.8)	273 (31.6)	0.100
Obese	170 (20.0)	159 (18.3)	190 (22.0)	

* Chi-square Test.

In the male group, we also noted that workers who had longer working hours were younger (average of 38.2 years) than those with short working hours (p < 0.001). In addition, night shift, greater number of jobs, and being outsourced were also significantly associated with longer working hours. Significant associations were observed between time working in nursing and working hours: nurses with average working hours (49.5 to 70.5 weekly) showed increased time working in nursing. Long working hours were associated with the absence of practice of physical activity. The proportion of nurses classified in the high strain group was higher among those who had long working hours than among those with short working hours (Table 2).

**Table 2:** Characterization of the weekly working hours of the male group according to socio-demographic, occupational, and health-related variables. Rio de Janeiro, State of Rio de Janeiro, Brazil, 2011.

Characteristics studied	Weekly working hours			p*

	Short (< 49,5h)	Average (49,5–70,5h)	Long (> 70,5h)	
Age (average, SD)	41.8 (11.4)	43.7 (10.2)	38.2 (9.5)	< 0.001
Marital status (n, %)				
Married/Common-law marriage	88 (33.6)	82 (31.3)	92 (35.1)	0.944
Single/Without partner	40 (32.8)	37 (30.3)	45 (36.9)	
Education level				
Post-graduate (*lato sensu* and *stricto sensu*)	82 (30.7)	88 (33.0)	97 (36.3)	0.271
University degree	45 (38.5)	31 (26.5)	41 (35.0)	
Household income *per capita* (n, %)				
Up to R$1,394.83	35 (27.8)	51 (40.5)	40 (31.7)	
R$1,394.90 to R$2,324.50	58 (34.1)	48 (28.2)	64 (37.6)	0.053
R$2,324.83 to R$7,440.00	26 (43.3)	13 (21.7)	21 (35.0)	
Hours on household chores (average, SD)	14.2 (13.7)	12.5 (11.5)	11.7 (10.7)	0.294
Time working in nursing (average, SD)	16.0 (11.6)	19.1 (10.1)	13.8 (9.6)	< 0.001
Work shift (n, %)				
Day	51 (35.2)	57 (39.3)	37 (25.5)	0.003
Night	76 (32.2)	62 (26.3)	98 (41.5)	
Number of jobs (n, %)				
One job	65 (79.3)	10 (12.2)	7 (8.5)	< 0.001
Two or more jobs	64 (20.8)	110 (35.8)	133 (43.3)	
Type of employment (n, %)				
Public servant	82 (33.6)	91 (37.3)	71 (29.1)	< 0.001
Outsourced	44 (32.1)	28 (20.4)	65 (47.4)	
Effort-reward imbalance (n, %)				
Low	48 (39.3)	44 (38.6)	32 (23.7)	
Average	38 (31.1)	33 (28.9)	49 (36.6)	0.053
High	36 (29.6)	37 (32.5)	54 (39.7)	
Demand-control (n, %)				
Low strain	49 (42.2)	38 (33.0)	32 (24.4)	
Passive work	14 (12.1)	27 (23.5)	19 (14.5)	0.008
Active work	29 (25.0)	27 (23.5)	36 (27.5)	
High strain	24 (20.7)	23 (20.0)	44 (33.6)	
Social support in the workplace (n, %)				
High	51 (41.1)	47 (40.5)	46 (33.3)	0.350
Low	73 (58.9)	69 (59.5)	92 (66.7)	
Thinking about leaving the profession (n, %)				
Not frequently	96 (75.6)	92 (76.7)	102 (72.9)	0.762
Frequently	31 (24.4)	28 (23.3)	38 (27.1)	
Practice of physical activity (n, %)				
Yes	64 (40.0)	49 (30.6)	47 (29.4)	0.028
No	65 (28.5)	70 (30.7)	93 (40.8)	
Duration of sleep per night (n, %)				
Up to 6.5h	59 (29.2)	63 (31.2)	80 (39.6)	
From 6.5 to 8.5h	49 (34.0)	46 (31.9)	49 (34.0)	0.410
From 8.5 to 12h	15 (44.1)	10 (29.4)	9 (26.5)	
Use of tobacco				
Smoker	10 (27.0)	13 (35.1)	14 (37.8)	
Former smoker	39 (51.3)	19 (25.0)	18 (23.7)	0.006
Non-smoker	79 (29.2)	86 (31.7)	106 (39.1)	
Use of alcoholic beverages (n, %)				
Never	40 (38.1)	27 (25.7)	38 (36.2)	
Up to 4 times a month	60 (28.0)	72 (33.6)	82 (38.3)	0.093
More than 4 times a month	28 (43.8)	19 (29.7)	17 (26.6)	
Body mass index (n, %)				
Eutrophic	41 (33.9)	37 (33.0)	33 (24.8)	
Overweight	53 (43.8)	51 (45.5)	70 (52.6)	0.505
Obese	27 (22.3)	24 (21.5)	30 (22.6)	

* Chi-square Test.

The bivariate analyses regarding the self-rated health showed common significant associations for the two groups, such as the effort-reward imbalance, high strain, the frequent thought of abandoning the profession, lack of physical activity, and obesity. In addition, the self-rated poor health of the women was also associated with short sleep at night and lack of social support at the workplace (Table 3). Among the men, there were significant associations with the number of jobs, and we observed that the self-rated regular health was more common among those who had two or more jobs. The persons who assessed their health as regular were younger, with shorter time working in nursing and they reported thinking more frequently about leaving the profession (Table 4).

**Table 3:** Characterization of self-rated health of the female group according to socio-demographic, occupational, and health-related variables. Rio de Janeiro, State of Rio de Janeiro, Brazil, 2011.

Characteristics studied	Self-reported health			p

	Good	Regular	Poor	
Age (average, SD)	40.0 (10.0)	39.5 (9.5)	38.9 (10.0)	0.227
Marital status (n, %)				
Married/Common-law marriage	999 (64.5)	437 (28.2)	113 (7.3)	0.279
Single/Without partner	824 (67.3)	314 (25.7)	86 (7.0)	
Education level				
Post-graduate (*lato sensu* and *stricto sensu*)	1,389 (66.0)	564 (26.8)	151 (7.2)	0.813
University degree	434 (64.8)	188 (28.1)	48 (7.2)	
Household income *per capita* (n, %)				
Up to R$1,394.83	671 (67.8)	256 (25.9)	62 (6.3)	
R$1,394.90 to R$2,324.50	815 (66.1)	332 (26.9)	86 (7.0)	0.191
R$2,324.83 to R$7,440.00	227 (61.2)	111 (29.9)	33 (8.9)	
Hours on household chores (average, SD)	20.9 (17.7)	22.4 (17.8)	22.5 (18.7)	0.119
Time working in nursing (average, SD)	15.3 (9.8)	15.0 (9.0)	14.7 (9.6)	0.647
Work shift (n, %)				
Day	755 (66.2)	302 (26.5)	83 (7.3)	0.836
Night	1,089 (65.5)	457 (27.5)	117 (7.0)	
Number of jobs (n, %)				
One job	659 (67.5)	253 (25.9)	64 (6.6)	0.346
Two or more jobs	1,185 (64.9)	506 (27.7)	136 (7.4)	
Type of employment (n, %)				
Public servant	1,196 (64.4)	526 (28.3)	136 (7.3)	0.118
Outsourced	595 (68.4)	220 (25.3)	55 (6.3)	
Effort-reward imbalance (n, %)				
Low	577 (35.6)	165 (24.7)	28 (15.3)	
Average	520 (32.1)	199 (29.7)	43 (23.5)	< 0.001
High	524 (32.3)	305 (45.6)	112 (61.2)	
Demand-control (n, %)				
Low strain	527 (30.6)	171 (24.1)	26 (13.6)	
Passive work	390 (22.7)	148 (20.9)	30 (15.7)	< 0.001
Active work	419 (24.4)	180 (25.4)	47 (24.6)	
High strain	384 (22.3)	210 (29.6)	88 (46.1)	
Social support in the workplace (n, %)				
High	991 (55.2)	340 (46.1)	81 (41.3)	< 0.001
Low	806 (44.8)	397 (53.9)	115 (58.7)	
Thinking about leaving the profession (n, %)				
Not frequently	1,511 (82.3)	576 (76.1)	118 (59.0)	< 0.001
Frequently	324 (17.7)	181 (23.9)	82 (41.0)	
Practice of physical activity (n, %)				
Yes	297 (36.2)	273 (33.3)	250 (30.5)	0.043
No	597 (31.9)	627 (33.5)	650 (34.7)	
Duration of sleep per night (n, %)				
Up to 6.5h	407 (32.2)	415 (32.9)	441 (34.9)	
From 7 to 8h	406 (35.9)	372 (32.9)	352 (31.2)	0.039
From 8.5 to 12h	79 (27.7)	109 (38.2)	97 (34.0)	
Use of tobacco				
Smoker	85 (36.3)	72 (30.8)	77 (32.9)	0.858
Former smoker	134 (33.4)	132 (32.9)	135 (33.7)	
Non-smoker	682 (32.9)	698 (33.7)	690 (33.3)	
Use of alcoholic beverages (n, %)				
Never	355 (34.0)	350 (33.5)	340 (32.5)	
Up to 4 times a month	452 (32.1)	473 (33.5)	485 (34.4)	0.489
More than 4 times a month	85 (37.3)	75 (32.9)	68 (29.8)	
Body mass index (n, %)				
Eutrophic	980 (55.9)	267 (37.1)	60 (31.5)	
Overweight	511 (29.2)	247 (34.3)	53 (27.7)	< 0.001
Obese	261 (14.9)	206 (28.6)	78 (40.8)	

* Chi-square Test.

**Table 4:** Characterization of the self-rated health of the male group according to socio-demographic, occupational, and health-related variables. Rio de Janeiro, State of Rio de Janeiro, Brazil, 2011.

Characteristics studied	Self-reported health			p

	Good	Regular	Poor	
Age (average, SD)	42.3 (10.5)	39.1 (10.8)	41.8 (10.7)	0.031
Marital status (n, %)				
Married/Common-law marriage	179 (65.1)	81 (29.5)	15 (5.5)	0.116
Single/Without partner	86 (67.2)	29 (22.7)	13 (10.2)	
Education level				
Post-graduate (*lato sensu* and *stricto sensu*)	191 (68.0)	69 (24.6)	21 (7.5)	0.168
University degree	73 (59.8)	42 (34.4)	7 (5.7)	
Household income *per capita* (n, %)				
Up to R$1,394.83	93 (68.9)	35 (25.9)	7 (5.2)	
R$1,394.90 to R$2,324.50	106 (61.6)	54 (31.4)	12 (7.0)	0.622
R$2,324.83 to R$7,440.00	43 (68.3)	15 (23.8)	5 (7.9)	
Hours on household chores (average, SD)	11.7 (11.0)	14.9 (13.7)	12.5 (12.9)	0.087
Time working in nursing (average, SD)	17.1 (11.0)	14.3 (0.8)	17.7 (10.7)	0.049
Work shift (n, %)				
Day	76 (73.1)	20 (19.2)	8 (7.7)	0.085
Night	192 (63.0)	93 (30.5)	20 (6.6)	
Number of jobs (n, %)				
One job	62 (72.1)	15 (17.4)	9 (10.5)	0.032
Two or more jobs	206 (63.8)	98 (30.3)	19 (5.9)	
Type of employment (n, %)				
Public servant	174 (68.4)	70 (27.1)	14 (5.4)	0.332
Outsourced	89 (63.1)	39 (27.7)	13 (9.2)	
Effort-reward imbalance (n, %)				
Low	108 (41.7)	18 (17.1)	6 (23.1)	
Average	80 (30.9)	38 (36.2)	6 (23.1)	< 0.001
High	71 (27.4)	49 (46.7)	14 (53.8)	
Demand-control (n, %)				
Low strain	99 (39.6)	21 (20.2)	5 (19.2)	
Passive work	47 (18.8)	15 (14.4)	4 (15.4)	< 0.001
Active work	61 (24.4)	30 (28.8)	6 (23.1)	
High strain	43 (17.2)	38 (36.6)	11 (42.3)	
Social support in the workplace (n, %)				
High	108 (41.4)	37 (33.9)	7 (27.9)	0.159
Low	153 (58.6)	72 (66.1)	20 (74.1)	
Thinking about leaving the profession (n, %)				
Not frequently	214 (80.1)	72 (63.7)	18 (66.7)	0.002
Frequently	53 (19.9)	41 (36.3)	9 (33.3)	
Practice of physical activity (n, %)				
Yes	133 (78.2)	31 (18.2)	6 (3.5)	< 0.001
No	133 (56.4)	81 (34.3)	22 (9.3)	
Duration of sleep per night (n, %)				
Up to 6.5h	126 (60.6)	61 (29.3)	21 (10.1)	0.060
From 7 to 8h	105 (68.6)	43 (28.1)	5 (3.3)	
From 8.5 to 12h	27 (75.0)	8 (22.2)	1 (2.8)	
Use of tobacco (n, %)				
Smoker	26 (65.0)	9 (22.5)	5 (12.5)	0.462
Former smoker	50 (63.3)	23 (29.1)	6 (7.6)	
Non-smoker	190 (66.9)	79 (27.8)	15 (5.3)	
Use of alcoholic beverages (n, %)				
Never	60 (55.0)	40 (36.7)	9 (8.3)	0.069
Up to 4 times a month	156 (70.0)	54 (24.2)	13 (5.8)	
More than 4 times a month	50 (71.4)	15 (21.4)	5 (7.1)	
Body mass index (n, %)				
Eutrophic	85 (33.5)	23 (22.1)	7 (26.0)	
Overweight	122 (48.0)	51 (49.0)	10 (37.0)	0.037
Obese	47 (18.5)	30 (28.9)	10 (37.0)	

* Chi-square Test.

In relation to the multivariate analyses, both in the male and the female groups, no significant associations were found between working hours and the category “self-rated poor health” (Table 5). Female nurses exposed to more than 60.5 hours per week (long working hours) were 43% more likely to assess their current state of health as regular when compared to those that had short working hours (up to 46.5 h/week). The association between exposure and outcome remained significant even after the adjustments in the multinomial logistic regression model (odds ratio [OR] = 1.30; 95%CI 1.02–1.67). Regarding the male sample, the adjusted odds ratio showed that workers exposed to intermediate working hours (49.5–70.5 hours per week) were 2.17 (95%CI 1.08–4.35) more likely to assess their health as regular than those with shorter working hours (< 49.5) after adjusting for the confounding variables. Those with working hours over 70.5 h/week tended to assess their health as regular; however, this relationship did not remain significant after adjusting for the confounding variables.

**Table 5:** Association between weekly working hours and self-rated health. Odds ratio (OR) and confidence interval (95%CI) based on multinomial logistic regression. Rio de Janeiro, State of Rio de Janeiro, Brazil, 2011.

Duration of working hours	n	Self-rated health^a^			

		Regular		Poor	
	
		_Crude_OR (95%CI)	_Adjusted_OR (95%CI)	_Crude_OR (95%CI)	_Adjusted_OR (95%CI)
Women^b^					
< 46.5h	900	1.0	1.0	1.0	1.0
46.5h–60.5h	903	1.17 (0.95–1.46)	1.06 (0.83–1.36)	1.01 (0.70–1.46)	0.92 (0.59–1.44)
> 60.5h	903	1.43 (1.16–1.77)	1.30 (1.02–1.67)	1.16 (0.81–1.67)	0.99 (0.63–1.55)
Men^c^					
< 49.5h	129	1.0	1.0	1.0	1.0
49.5–70.5h	120	1.80 (1.00–3.25)	2.17 (1.08–4.35)	0.68 (0.22–2.10)	0.46 (0.12–1.72)
> 70.5h	139	2.34 (1.32–4.13)	1.85 (0.93–3.66)	1.76 (0.71–4.33)	1.13 (0.38–3.32)

^a^ Comparisons regarding good self-rated health.

^b^ Adjusted OR for: income, marital status, age; work shift, job, effort-reward imbalance; sleep, and physical activity.

^c^ Adjusted OR for: income, marital status, age; work shift, job, effort-reward imbalance; physical activity.

## DISCUSSION

The results partially confirm the hypotheses of the study. Among women, the adjusted data indicate greater chance of regular health among those with longer working hours (at least 60.5 h/week). Among men, the chance for regular health was twice as likely in the group with intermediate working hours (49.5–70.5 h/week) than whose working less than 49.5 hours per week.

It is worth mentioning that the classification of working hours is based on the profile of the group studied, and we emphasize that even the intermediate working hours considered herein already implies an excessive working time when compared to that observed in other countries. Such differences in the length of the working hours hinder the comparison with results of other authors, as noted by Van der Hulst^29^. According to the author^29^, some studies, mainly with Japanese workers, refer to extremely long working hours, so that the reference group includes persons who work more than 40 h/week and who, therefore, could present health problems resulting from long working hours. In this study, according to the working hours analyzed, it is possible that the observed associations have been underestimated, when compared to a reference group with shorter working hours.

In this investigation, the average weekly working hours – 55.0 and 61.0 h/week for women and men, respectively – confirm previous results with nursing professionals, which also indicate extensive working hours from the common practice of having two or more jobs^22^. In fact, although most hospitals studied adopt a system of shifts named 12/60 (12-hour shifts followed by 60 hours off), which entails 30 working hours weekly, only 1/3 of the sample works in only one location^10^. The long working hours observed in this study contrasts with the values observed in another study^13^ with nurses from fifteen European countries, whose average working hours ranged from 24.5 (Netherlands) to 38.5 h/week (Slovakia).

Our results remind the review conducted by Caruso et al.^4^, in which the authors have observed that extensive working hours (> 40 h/week) were associated with diseases and mortality data, in addition to greater alcohol consumption. Another review^29^ highlights that exposure to long working hours is reflected in physiological (decreased immune response) and behavioral changes (reduced hours of sleep). Recent data also indicate adverse effects of extensive working hours in relation to sleep duration and quality^17^. In fact, data from the literature report the association between long working hours and higher prevalence of metabolic syndrome and weight gain, as well as a higher incidence of coronary heart disease and depressive symptoms^2^.

The possible mechanisms and pathways by which long working hours could affect health fall into two main aspects^4^: (i) lower availability of time for sleep and recovery, as well as for family and leisure activities and (ii) greater exposure or increased vulnerability to demands and risks from work. In the case of nursing staff, the first aspect can be exemplified by a qualitative study^19^ that has shown that nurses assign their illness to work overload and lack of care arising from the excessive time devoted to professional work.

Regarding the greater exposure to the demands of the work (second aspect), it is known that the nursing staff is usually exposed to unhealthy environments from the material point of view, which is combined with high emotional distress^7^. In this study, long working hours were associated with several occupational factors known to be harmful to health, such as outsourced work, effort-reward imbalance, and psychosocial distress assessed using the demand-control model. In this regard, a large study involving more than 500 American hospitals has noted that, the longer the working hours, the greater the level of burnout of nurses and the dissatisfaction of patients^27^. Therefore, there are various elements of the organization and the work environment that may influence the relationships between long working hours and the health of workers.

The separate analyses for the male and female samples is due to data from literature on gender differences both in relation to self-rated health^15^ and in relation to working hours^9,22^. In addition, the demand for stratified analyses in this study is evidenced in a research conducted by Song et al.^26^ The study^26^ has described the increase of more than 40% in the classification of poor health among workers (men and women in various professions) with working hours over 60 h/week, compared to those with less working hours (up to 40 h/week). However, after observing a significant association in the mixed sample, the stratification according to gender showed a significant association only in the female sample^26^. Study on excessive working time and symptoms of depression in mixed samples has also detected significant associations only among women^30^. Together, these results reinforce the statement of Van der Hulst^29^, according to whom there is a central gap in the analysis of possible gender differences in the relationship between working hours and health that needs to be addressed in studies on this subject.

The similarity between the male and female samples regarding self-rated health differs from previous studies, which usually show worse health condition among women^5^. We did not identify, in the samples studied, characteristics that explain such results. In addition, the two samples were also similar regarding some bivariate analyses, as in both the self-rated health was associated with psychosocial stress, assessed using two independent scales, thinking about leaving the profession, lack of physical activity, and body mass index. In this case, the associations observed are plausible from the abundant literature on the relationships between health and the aforementioned variables (psychosocial environment at work, physical activity, and obesity) both between men and women^1^. A previous study with nurses from various countries has observed the relationship between the thought of leaving the profession and the health of workers^12^.

A similar profile was also observed between men and women exposed to long working hours. They are the groups that included a higher proportion of younger persons, who were in the profession for less time, outsourced, working at night, classified in the category of high strain (high demand and low control), and who did not practice physical activity. We highlight other characteristics observed only in the female sample: higher proportion of workers with short sleep duration (less than 6.5 hours/day), high effort-reward imbalance, low social support, and who reported frequently thinking about leaving the profession. These results express the current situation of deterioration of the work-health relations, in particular regarding the female sample. In fact, in Brazil, the 12-hour shifts together with the accumulation of jobs are a reality among nurses, who can do 24-hour shifts in some hospitals^21^. This situation shows the demand for interventions in the organization of the nursing work, including aspects related to the number of jobs, which will benefit not only the health of workers, but also the quality of the care to patients^27^. Regarding the profile of workers subjected to long working hours, we can mention the discussion on the work as a source of illness, emphasizing the role of insufficient sleep combined with poor recovery as a common pathway that would connect the long working hours, shift work, and the occupational stress with the health of workers^11^.

In this investigation, significant associations in the adjusted data refer exclusively to self-rated regular health. The low percentage of workers who assessed their health as poor – approximately 7% – was noted earlier. In a study with employees of an industry, only 0.5% of the workers assessed their health as poor^3^. The low percentage of poor self-rated health possibly arises from the healthy worker effect^18^, in the sense that workers in worse health conditions are no longer active and, therefore, would not have been included in the eligible group. This is an inevitable bias in cross-sectional studies in the field of occupational health.

Some limitations may have influenced the results identified. Among them, the impossibility to establish the temporality relationship between working hours and self-rated health hinders the establishment of causal relationships between exposure and outcome. Although we have tested different variables as potential confounding ones, we cannot ruled out the possible influence of other factors not covered in the study, such as data on mental health and specific characteristics of the nursing work (such as the relationships with patients and family members). The study involved self-reported information, which can lead to information measurement biases. Seeking to minimize such biases, the process to obtain the data had strict control to ensure the quality of the data. Among the activities to control the quality of the data, we highlight the training and standardization of the field staff, the development of manuals for the procedures of the team, and the review of the questionnaires filled out when collecting them.

Although presenting limitations, the self-reported information represents an alternative to clinical measures in health assessment in epidemiological studies^20^. Although the calculation of weekly working hours based in the recall of the past seven days is one of the strengths of the research, the data may not represent the work exposure throughout the working life. Another aspect that deserves mention is the sample. Nurses have particular characteristics that combine work in shifts and multiple jobs in environments considered as stressful from the emotional point of view^7^. In addition, the relatively small size of the male sample may have reduced the statistical power of the analyses. In this sense, the findings should not be generalized to other professional categories.

It is worth noting, as a positive point of the study, the use of real time dedicated to professional work, as recommended by another study^7^. This information was obtained by the recall of the hours worked daily throughout the week, which gives a more real dimension of the variable of exposure, when compared to information from human resources services. This aspect is particularly relevant in the case of nursing teams, given the flexibility to change shifts between professionals^21^. Another positive point refers to the stratification of the analyses according to gender, which allowed us to detect relevant differences in the results.

The complexity of the relationships between working hours and health is emphasized by several authors, given the influence of a range of occupational factors (psychosocial aspects such as demands, rewards, social support, control over work and working hours, and shifts, among others) and individual variables, such as socio-demographic profile and household chores^4,11^. In this context, the associations observed in this study were detected even after adjusting for several variables, which suggests a consistent relationship between excess hours of work and the view of workers about their own health.

In short, considering that the self-rated of health consistently expresses aspects of morbidity and mortality^28^, the results expose the urgency for actions aimed at the appreciation of the profession, in particular regarding the current situation of multiple jobs, which implies excessive working hours with possible repercussions on the health of workers and the quality of care in hospitals.
